# Simultaneous genome-wide gene expression and transcript isoform profiling in the human malaria parasite

**DOI:** 10.1371/journal.pone.0187595

**Published:** 2017-11-07

**Authors:** Lindsey B. Turnbull, Geoffrey H. Siwo, Katrina A. Button-Simons, Asako Tan, Lisa A. Checkley, Heather J. Painter, Manuel Llinás, Michael T. Ferdig

**Affiliations:** 1 Department of Biological Sciences, Eck Institute for Global Health, University of Notre Dame, Notre Dame, Indiana, United States of America; 2 Ryan White Center for Pediatric Infectious Diseases and Global Health, Indiana University, Indianapolis, Indiana, United States of America; 3 IBM Research Africa, Johannesburg, South Africa; 4 Illumina, Madison, Wisconsin, United States of America; 5 Department of Biochemistry & Molecular Biology and Center for Malaria Research, Pennsylvania State University, University Park, Pennsylvania, United States of America; 6 Department of Chemistry, Pennsylvania State University, University Park, Pennsylvania, United States of America; Florida Atlantic University, UNITED STATES

## Abstract

Gene expression DNA microarrays have been vital for characterizing whole-genome transcriptional profiles. Nevertheless, their effectiveness relies heavily on the accuracy of genome sequences, the annotation of gene structures, and the sequence-dependent performance of individual probes. Currently available gene expression arrays for the malaria parasite *Plasmodium falciparum* rely on an average of 2 probes per gene, usually positioned near the 3′ end of genes; consequently, existing designs are prone to measurement bias and cannot capture complexities such as the occurrence of transcript isoforms arising from alternative splicing or alternative start/ stop sites. Here, we describe two novel gene expression arrays with exon-focused probes designed with an average of 12 and 20 probes spanning each gene. This high probe density minimizes signal noise inherent in probe-to-probe sequence-dependent hybridization intensity. We demonstrate that these exon arrays accurately profile genome-wide expression, comparing favorably to currently available arrays and RNA-seq profiling, and can detect alternatively spliced transcript isoforms as well as non-coding RNAs (ncRNAs). Of the 964 candidate alternate splicing events from published RNA-seq studies, 162 are confirmed using the exon array. Furthermore, the exon array predicted 330 previously unidentified alternate splicing events. Gene expression microarrays continue to offer a cost-effective alternative to RNA-seq for the simultaneous monitoring of gene expression and alternative splicing events. Microarrays may even be preferred in some cases due to their affordability and the rapid turn-around of results when hundreds of samples are required for fine-scale systems biology investigations, including the monitoring of the networks of gene co-expression in the emergence of drug resistance.

## Introduction

Even as RNA sequencing (RNA-seq) studies become common, microarrays remain the most widely used tool to assess gene expression patterns [1]. This preference for microarrays for studies containing large sample numbers is partly due to the lower cost; furthermore, microarray data can be generated faster due to well-established data analytical tools and pipelines. Moreover, it can be more straightforward to combine data and compare results across projects and labs when using microarrays [2]. However, for good technical reasons, RNA-seq is superior, and if microarray technology is to survive, array platforms need to be updated to take advantage of all potential applications. Specifically, the dynamic range of microarrays does not approximate that of RNA sequencing when samples are sequenced at high enough coverage levels, which vary based on the size of the transcriptome, and the number of lowly expressed genes [3,4]. Microarrays are generally not well-suited to identify transcript isoforms—an important biological feature of gene expression that remains to be fully explored.

For *Plasmodium falciparum*, the most lethal of malaria species, gene regulatory control of the parasite across the developmental cycle remains an active area of research. Transcription is an essential layer of information to guide the understanding of how phenotypes arise and change over time, across genetic variation, and in response to changes in the environment. Gene expression arrays have provided a relatively unbiased technique to quantify genome-wide transcript levels at high resolution, demonstrating that gene expression is regulated throughout the developmental cycle [5–7], that the genome contains hotspots for transcriptional regulation [8], and that expression patterns change in response to drugs and other perturbations [9–12]. Gene expression profiling of *in vivo* parasite samples has demonstrated that the parasite exists in three distinct physiological states each with implications for disease severity [13,14]. Recently, whole transcriptome data have revealed differences in transcriptional networks between drug sensitive and resistant parasites [15] as well as potentially novel mechanisms for anti-malarial drugs [16,17]. A high-density microarray for *Plasmodium falciparum* recently was used to demonstrate the affordability and feasibility to predict drug mechanisms of action further emphasizing the continuing usefulness of this technology [17]. Given the amount of data generated in each of these studies, the comparison of gene expression across hundreds of samples and different studies may hold the key to understanding the significance of gene expression in contributing to a myriad of biological phenotypes.

In spite of the proven utility of gene expression arrays, current microarray platforms for *P*. *falciparum* face a number of challenges that confound their ability to capture biological variation. Previously utilized Affymetrix arrays interrogated the transcriptome with a high density of approximately one probe per 150bp [16,18], though exact probe placement is not uniform across the transcriptome. These arrays are designed such that both detection of ncRNAs and alternative splicing is possible. However, binding to the 25 base pair length oligonucleotide probes on these arrays is less specific and has higher potential cross reactivity than more recent glass slide arrays that use longer probes that are either spotted or synthesized *in situ*. Affymetrix arrays only measure a single sample per array leading to a high cost per sample, and redesigning Affymetrix arrays is expensive, thus making it cost prohibitive to keep it up to date with the reference genome. Newer, multiple sample platforms with design flexibility are thus more feasible for studies with large sample sizes. Two microarray platforms currently in use include a spotted oligonucleotide array with 70bp probes with about 1.9 probes per gene [5,7], and an Agilent 8×15K array with 60mer probes and average of 2.5 probes per gene [19,20]. The Agilent 15K array is a significant improvement over previous arrays in terms of number of features represented including non-coding RNAs (ncRNAs) and recently identified genes. As a dual-color platform, the Agilent 15K array (with 2.5 probes per gene on average) adequately captures the log_2_ ratio of co-hybridized samples which have been shown to be statistically consistent regardless of probe number and location within a gene [19,20]. However, overall signal intensity for a given probe within a gene on the Agilent 15K array is highly variable [19,20] based on nucleotide sequence [21,22], and an average of 2.5 probes per gene could lead to biases in average gene expression levels that can be strongly influenced by outliers and sequence polymorphisms. Given the highly sequence-dependent nature of probe hybridization kinetics [21,22] average gene expression levels would be expected to be less biased if expression levels were obtained from the signal intensities of multiple probes; this is especially relevant given the highly AT rich genome of *P*. *falciparum*. In addition, nearly half of the genes in *P*. *falciparum* contain multiple exons [23] and the potential for transcript isoforms. Current microarray platforms cannot resolve transcript isoforms arising from alternatively spliced exons, alternative promoters or alternative start and stop sites. Genome-wide characterization of alternative splicing has not been conducted using microarrays; however, two RNA-seq studies predicted that at least 4.5% of genes undergo alternative splicing in *P*. *falciparum* across developmental time points [24,25]. Another study, based on cDNA sequencing of EST libraries, estimated that nearly 16% of genes are alternatively spliced [26].

To enhance the information attainable from microarrays, we designed and validated two high density microarrays, one on the Nimblegen platform and one on Agilent’s high density (HD) 60K platform. The Nimblegen array was first described in a 2015 publication [17] and is validated in greater detail here. Both of these arrays simultaneously measure genome-wide transcript levels and transcript isoforms. As has been done in other organisms, alternatively spliced variants are determined by leveraging multiple probes spanning a single gene [27–29]. Probe sets were designed to target each exon and alternative splicing was determined by comparing the signal intensity of a given exon to the average intensity of all probes in the same gene [27]. In contrast to existing *Plasmodium* arrays, both of these exon-centric arrays contain multiple probes per exon and a large number of overall probes spanning each gene, conferring a new level of robustness which is less sensitive to probe-specific signal noise and allows both arrays to be used as single-color platforms. While the Nimblegen array has significantly more features per sample, this array is no longer available because Nimblegen has discontinued microarray production which prompted the design of the Agilent HD exon microarray.

## Results

We designed two exon expression microarrays to profile the full transcriptome of the malaria species *Plasmodium falciparum*. Both of these arrays have a substantially higher density of probes per replicated sample loading region (plex) on the chip than previous designs (Table 1), which allows for more precise measurement of expression at the exon level. The Nimblegen platform chip is a 12-plex microarray containing an average of 5 probes per exon and 22 per annotated gene (PlasmoDB v6.3). The Agilent HD exon array is an 8-plex microarray containing an average of 5 probes per exon and 12 per annotated gene (PlasmoDB v9.3). Both arrays also contain probes that interrogate 92 non-coding RNAs (ncRNAs) [18]. To compare the performance of our exon arrays with that of existing arrays we used data generated from the established Agilent 15K array [19] as the gold standard. Both the Nimblegen and Agilent HD exon array are utilized as a single-color platform for the data presented, while the Agilent 15K array is dual-color. Additional information about microarray specifications of our arrays and other existing *Plasmodium* microarrays is provided in Table 1.

**Table 1:** Exon array designs.

Array	Agilent HD Exon Array	Nimblegen Exon Array	DeRisi [5]	Bozdech [30]	Llinas [19]	Das [31]
**Platform**	Agilent	Nimblegen	Printed	Printed	Agilent	Agilent
**Plexes**	8	12	1	1	8	8
**Unique Probes**	62976	128179	7642	10159	14353	6362
**Range of Probes Per Exon**	1–52	5–20	N/A	N/A	N/A	N/A
**Average Probes Per Gene**	12	22	2	2	3	1.1
**Genes Represented**	5440	5683	4408	5363	5752	5276
**Transcript Isoform Profiling**	Yes	Yes	No	No	No	No
**ncRNAs**	Yes	Yes	No	No	Yes	Yes

Comparison of Nimblegen and Agilent HD Exon Arrays and Other Glass Slide *P*. *falciparum* Gene Expression Microarrays.[5,19,30,31]

**Fig 1:**
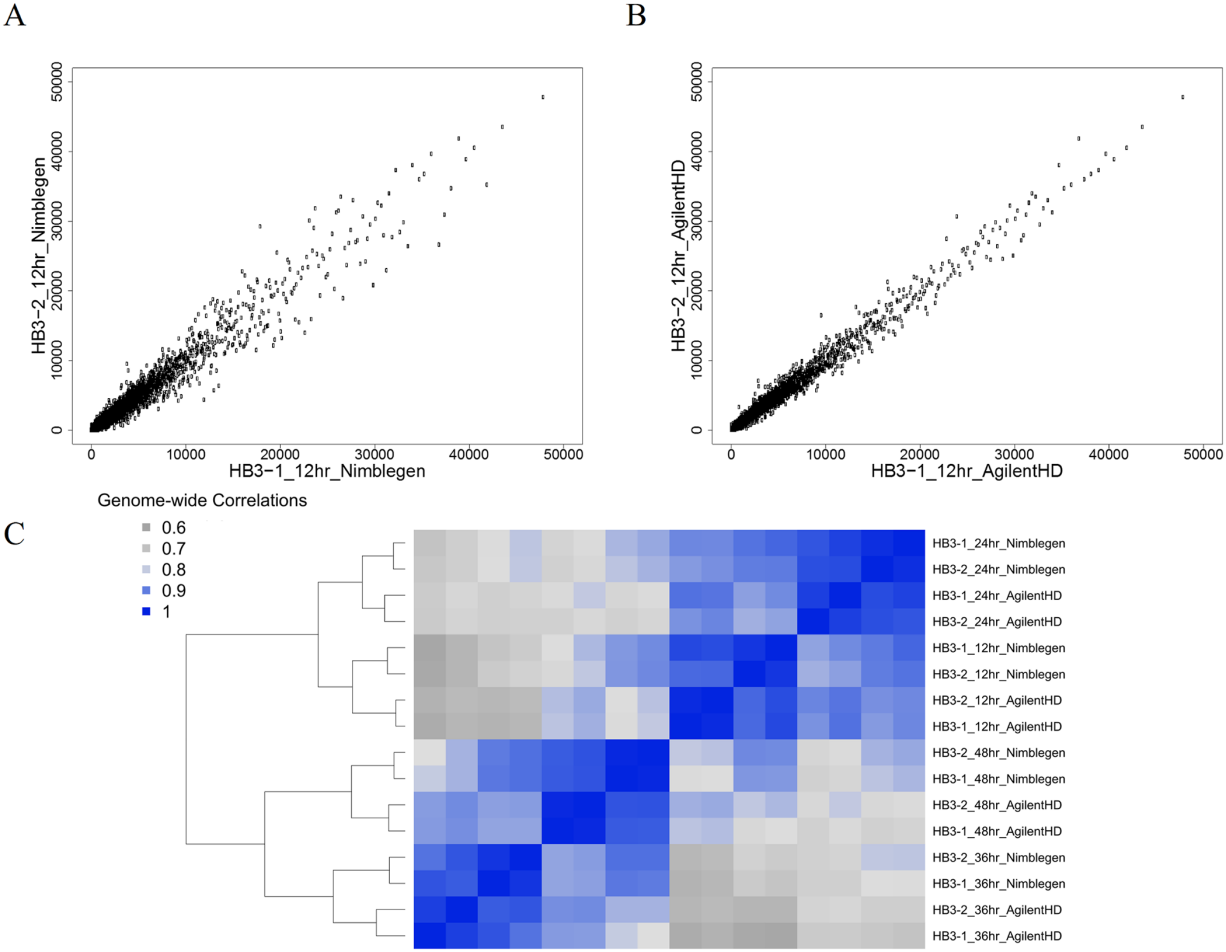
Biological replicates show high reproducibility on the exon arrays. Spearman correlations of biological replicates show highly reproducible signal for 12 hpi samples hybridized to the (A) Nimblegen and (B) Agilent HD exon arrays. (C) Hierarchical clustering of correlation values across all samples hybridized to the high-density exon arrays show reproducibility of signal between chip platforms across the intra-erythrocytic lifecycle.

To determine the within platform reproducibility of gene expression measurements obtained from our exon arrays, we hybridized independently biologically replicated RNA samples of the HB3 laboratory clone from 12, 24, 36, and 48 hours post-invasion (hpi). Both arrays generated reproducible whole transcriptome measurements at 12 hpi (Fig 1A and 1B), as well as at 24, 36 and 48 hpi (not shown). The correlations among replicates on the Nimblegen platform ranged from 0.96 (48 hpi) to 0.99 (12, 24, 36 hpi) (Fig 1C). For the Agilent HD exon array, the correlations observed between biological replicates were 0.96 (24, 36 hpi) and 0.99 (12, 48 hpi) (Fig 1C). Hierarchical clustering of samples by time point, irrespective of which array was used (Fig 1C), confirm that whole-transcriptome profiles are stable and robust across these two platforms. Correlation values observed between the same samples hybridized to the Agilent HD exon array and Nimblegen exon array (0.917 to 0.962, Fig 1C). This analysis demonstrates that both the Nimblegen exon array and Agilent HD exon array obtain highly reproducible mRNA abundance measurements on a single-color platform. Given the high correlations across biologically replicated samples, this data suggests that there may be greater experimental value in prioritizing biological replication over technical replication if costs are limiting.

### Differential gene expression reproducibility

**Fig 2:**
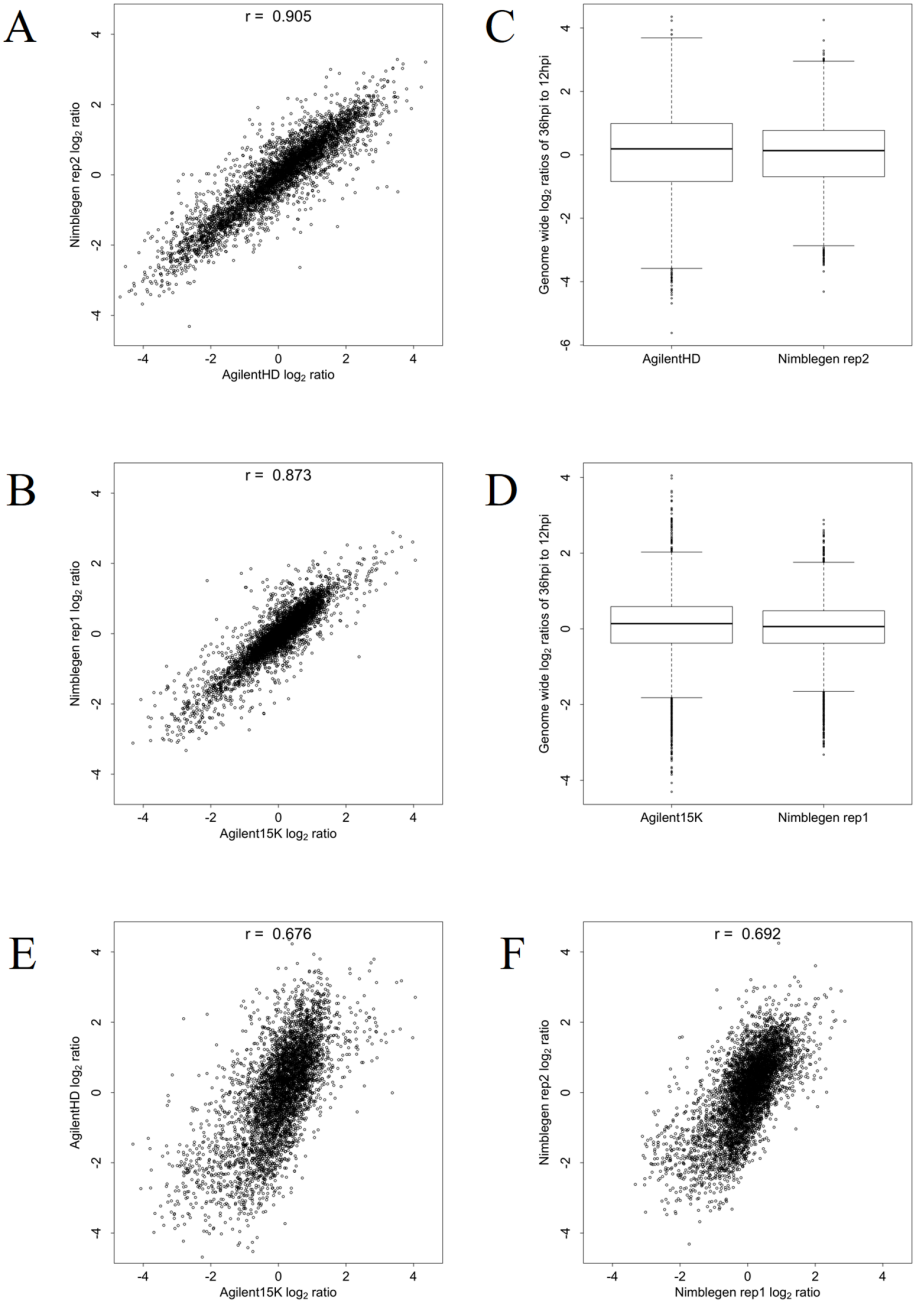
Differentially expressed genes are replicated with high concordance on the exon arrays. Log_2_ ratios of 12 hpi vs. 36 hpi samples were calculated to determine differentially expressed genes. The correlations between log_2_ ratios on the Nimblegen and Agilent HD exon (0.905, A and C) and Agilent 15K arrays (0.873, B and D) show that biologically relevant information is replicated across array platforms. The mean differential expression values are comparable between chip platforms, though both Agilent arrays have an overall larger dynamic range (C and D). The correlation between the Agilent 15K and Agilent HD exon array is 0.676 (E). Different biological samples were hybridized to these two arrays and the correlation value matches that of these two biological replicates when hybridized on the same platform (F, 0.692) suggesting that the variation seen is due to differences in the samples.

To determine the accuracy of the mRNA abundance captured on the exon arrays, we measured differential gene expression of biologically replicated HB3 samples between 12 and 36 hpi. Using an initial log_2_ = 2 (4-fold) differential expression cut-off to generate gene lists for comparing platforms, we identified up- and down-regulated candidate genes between these time points for the 4911 genes represented on both arrays. The correlation of genome-wide log_2_ ratios between the two exon arrays was 0.905 (Fig 2A and 2B). For the Nimblegen array we observed 92 up-regulated and 244 down-regulated genes. For the Agilent HD exon array we observed 348 up-regulated and 488 down-regulated genes (S1 and S2 Tables). Combined, both arrays shared 75 up-regulated genes and 219 as down-regulated, representing an average concordance of 85.6% between the array platforms with respect to up- or down-regulated genes. The majority of additional differentially expressed genes identified on the Agilent HD exon array are due to the higher signal:noise ratio of the Agilent array and the greater dynamic range of the Agilent scanner. When comparing each of these arrays to the data from the Agilent 15K array, the genome-wide log_2_ ratio correlations for the Nimblegen array was 0.873 (Fig 2C and 2D) with an average concordance of 80.09%. The overall correlation between the Agilent 15K and Agilent HD exon array was 0.676 (Fig 2E); the average concordance of up- and down-regulated genes between the Agilent arrays was 86.44%. This indicates that the lower correlation value does not indicate lower reproducibility of information between arrays. Rather, the lower correlation value can be largely attributed to differences in biological samples used on these two arrays, as the correlation between these biological replicates on the Nimblegen array to which both sets of samples were hybridized matches that observed on the corresponding Agilent array (0.692, Fig 2F). These comparisons show that the *P*. *falciparum* exon arrays capture gene expression patterns consistent with previously used array designs.

### Gene set enrichment

**Fig 3:**
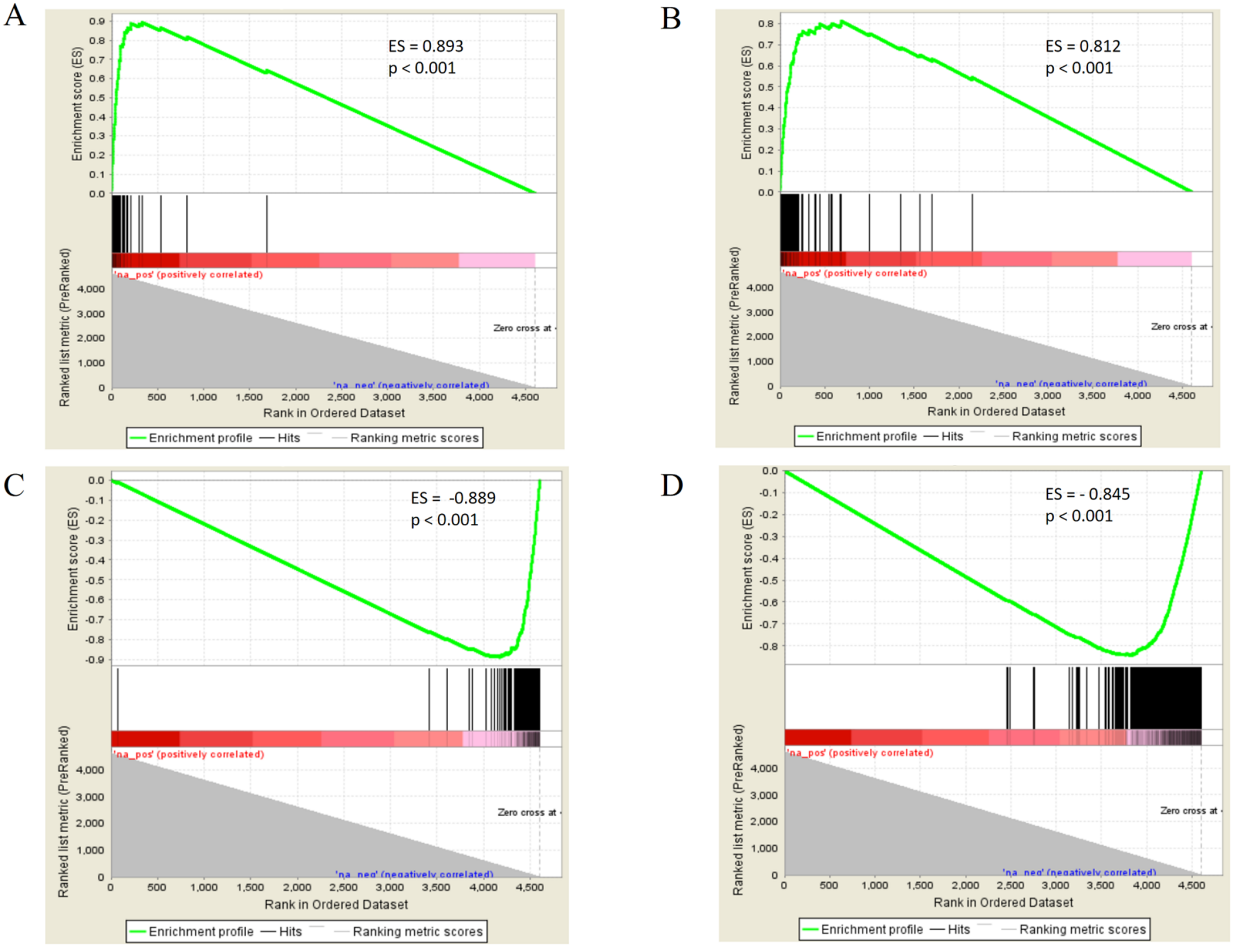
Gene set enrichment analysis (GSEA) of up-regulated genes between exon arrays and previously published 15K agilent array show high reproducibility of biological information. GSEA plots for chip to chip comparisons of unregulated genes for HB3 12 hpi vs 36 hpi samples. Black lines indicate the location of “hits” from the rank order list of the up or down-regulated genes from the query list. Correlation values between ranked ordered and query list is progressively calculated and indicated by the red bar (with stronger correlations indicated by darker hue). The “zero crosses at” dotted line indicates when the enrichment score decays back to zero and there are no more potential matches between the query and rank ordered lists. (A and C) Comparing the top up-regulated and down-regulated genes on the Agilent 15K array to the rank ordered list of all genes on the Nimblegen exon array. (B and D) Comparing the top up-regulated and down-regulated genes on the Agilent HD exon array to the rank ordered differential expression of all genes on the comparably designed Nimblegen exon array. These comparisons show that the top up-regulated genes between 12 hpi and 36 hpi are consistent across all 3 platforms.

Recognizing the problem with using arbitrary cut-offs in generating gene lists, and to further assess reproducibility and biologically meaningful information measured by our newly designed exon arrays, we performed Gene Set Enrichment Analysis (GSEA) [32] with the up-regulated and down-regulated genes from the Agilent 15K and Agilent HD exon array as query lists. The rank ordered list of genes on the Nimblegen array was arranged by decreasing log_2_ expression values for 12 vs. 36 hpi. The upper end of this ranked list was significantly enriched with genes from the corresponding up-regulated Agilent array gene set (Fig 3A and 3B, enrichment scores = 0.89 and 0.82, p < 0.001), while the lower range was significantly enriched with genes from the corresponding down-regulated Agilent array query lists (Fig 3C and 3D, enrichment scores = -0.89 and -0.85, p < 0.001). Together, these observations further confirm that the platforms are highly concordant and replicate biologically meaningful differences in gene expression between samples.

### Expression of ncRNAs

ncRNAs are increasingly recognized to play pivotal regulatory roles in many organisms; however, limited information is available on their role in *P*. *falciparum*. Tools for precise and cost-effective measurement of genome-wide ncRNAs levels, in concert with coding mRNA, will shed light on their role in parasite biology. The Nimblegen array includes 92 ncRNAs from a published set of 120 ncRNAs [18] that have been confirmed by northern blots, microarrays or phylogenetic conservation. The Agilent HD exon array includes 100 ncRNAs from the same set of verified ncRNAs. ncRNAs were only excluded based on the inability to design appropriate probes on each array platform. An important feature of the Agilent chip construction is the ease with which new information can be incorporated into future designs, for example as more regulatory ncRNAs are discovered these can be added to subsequent chips.

In HB3, 75 ncRNAs were detected in at least one developmental stage on the Nimblegen exon array, and 68 on the Agilent HD exon array (S3 Table). The Spearman correlation between the 51 ncRNAs detected on both arrays at 24 hpi was high (r = 0.82). Of 22 ncRNAs previously reported as expressed based on northern blots, 17 were represented on the Agilent HD exon array. Six of these ncRNAs were differentially expressed (2-fold change) between stages (S4 Table). Differentially expressed ncRNAs included RNAZ_ID_2132 expressed in rings and trophozoites, which exhibited a transcript level 4.5-fold lower in 36 hour early schizonts compared to 12 hour rings.

### Transcript isoforms

A single gene can encode more than one protein product and/or transcript isoform due to alternative splicing, including alternative transcription start or stop sites and alternative polyadenylation sites [33]. Exon arrays allow for the detection of transcript isoforms based on the expression level of individual exons across a gene [27,34]. An alternatively spliced exon (absent in some cells under some conditions) is expressed at a lower level compared to the expression level of the entire gene [27]. To test the ability of the exon array to detect previously described transcript isoforms, we obtained exon-level expression for each gene by averaging the signal intensity across all probes targeting an exon. The exon-level expression was then normalized to the average signal intensity across a gene to obtain the gene level normalized intensity (NI). Alternative splicing for a given exon was then determined as the log_2_ ratio of NI between two samples, referred to as the Splicing Index (SI) [27]. An exon was considered as a candidate for alternative splicing if it had an SI more than 2 or less than -2. Prior RNA-seq studies excluded highly variable genes (*var*, *rifin*, stevor) from data analysis of isoforms; for ease of comparison these genes were also trimmed from our lists of alternatively spliced genes.

**Fig 4:**
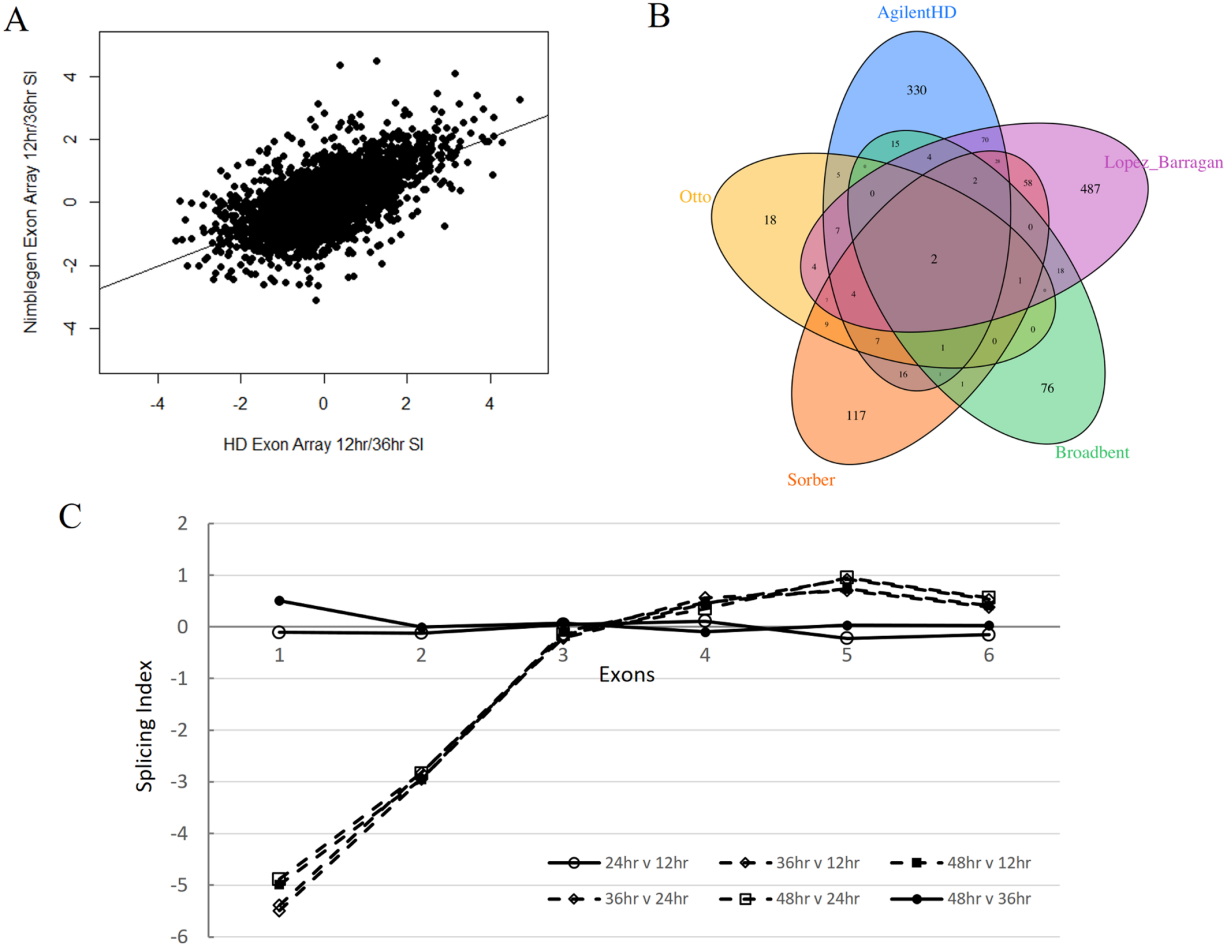
Exon arrays can detect exon skipping transcript isoforms. Comparison of genes with transcript isoforms (A) Differential exon splicing is detected on both Nimblegen and Agilent HD exon array platforms using splicing index with a correlation of 0.626. (B) Venn Diagram showing the overlap of genes with transcript isoforms reported by the Agilent HD Exon Array (blue) and RNA-seq studies (Otto (yellow), Sorber (orange), Broadbent (green), and Lopez-Barragen (purple) [24,25,36,37]), (C) Alternative start sites for PF3D7_0929200 produce time point dependent exon skipping of exons 1 and 2 during the schizont parasite stage at 36 and 48 hpi.

On this basis, we predicted 665 splicing events involving 492 genes in at least one pair of time point comparisons (S5 Table). The correlation of SI between our two exon arrays was 0.626 for the 12 hpi vs. 36 hpi time points (Fig 4A). This value is lower than the correlation observed across arrays for differential expression of the same time point, and is primarily due to the higher sensitivity of the splicing index to variation in expression estimate and is comparable to the between array SI comparison values on other platforms [35].

The number of alternative splicing events in *P*. *falciparum* has been estimated by RNA-seq studies to range from sixty-five [25] to six-hundred ninety-two [37] genes having at least one alternatively spliced product. To assess the performance of the exon array, we compared the predicted alternative splicing events to those from four RNA-seq studies: Otto *et al*. [25] in which alternative splicing was determined using sequence data from 3D7 parasites at 8, 16, 24, 32, 40 and 48 hpi, Sorber *et al*. [24] in which sequencing was performed on 11, 22, 33, and 44 hpi samples from the 3D7 laboratory clone; Broadbent *et al*.[36] who sequenced a total of 15 time points across the lifecycle and focused primarily on long noncoding RNA structure and splicing; and Lopez-Barragan *et al*.[37] which included sequencing of gametocytes and mosquito stages of the parasite lifecycle. Each of these studies used the 3D7 parasite strain, and harvested RNA at similar time points in the lifecycle. However the lists of alternatively spliced genes in these RNA-seq studies are largely exclusive such that each study identified a large number of unique genes with alternative splicing. Specifically, Otto *et al*. detected 65 genes with at least one alternative splicing event, Sorber *et al*. detected 254 genes, and the Agilent HD exon array predicted 523 S5 Table). Thirty-one out of 65 (48%, enrichment = 10.2 fold, hypergeometric p < 0.001) genes detected by Otto were also detected by Sorber. Similarly, 26 out of 65 genes (40%, enrichment = 4.42 fold, hypergeometric p < 0.001) detected by Otto were also detected on the exon array (Fig 4B). Sixty-one of the 254 (24%, enrichment = 2.65 fold, p <0.001) genes identified as alternatively spliced by Sorber were detected on the exon array. Only four and eight genes identified by Otto and Sorber respectively were among those cited as alternatively spliced by Broadbent. One hundred seventeen genes detected on the exon array were also identified in Lopez-Barragan’s analysis (22.3%, enrichment = 1.87 fold, p <0.001). Consequently, a total of 162 genes with transcript isoforms (32.9%) identified by the Agilent HD exon array were independently supported by RNA-seq data. The exon arrays also detected 330 potentially alternatively spliced genes not previously identified by other studies. Identification of novel isoforms on the microarray is not unexpected, and there are a large number of genes with suggested alternative splicing that are reported in only one of the four comparison RNA-seq studies (698 of 964 total). Of the 5668 annotated genes in the current PlasmoDB release 28, the exon array covers a total of 5434 (95.8%), and 4569 (80.6% total) genes are covered by eight or more unique probes. Alternative splicing could potentially be identified on 2781 genes (51.1%) represented on the HD exon array which have probes in at least two exons, though not every multiple exon gene is expected to have alternate transcripts.

Most transcript isoform candidates identified on the HD exon array were alternative transcription start and stop sites. For example PF3D7_0929200, coding for a putative RNA-binding protein, contains an alternative 3′ start site [25] that results in different mRNA sequence based on hpi time points (Fig 4C). The gene expresses two different transcript isoforms, one containing six exons at 12 and 24 hpi and the other with four at 36 and 48 hpi. Alternative splicing can lead to protein isoforms with different biological functions when the alternatively spliced exon encodes distinct protein domains. Therefore, for each spliced gene, we scanned the encoded protein sequence for domains using position specific scoring matrices (PSSMs) of protein domain alignments from the conserved domain database (CDD) [38]. Of the 492 alternatively spliced genes detected by the Agilent HD exon array, 29% (196 exons) coded for conserved protein domains from distinct functional categories such as enzymes, sorting signals, protein-protein interactions, protein-DNA interactions, transport domains, and variant surface antigens (S5 Table). Alternative splicing also can lead to changes in localization of proteins in the cell through splicing events involving exon coding for protein sorting signals or trans-membrane domains. In *P*. *falciparum*, an established example is erythrocyte binding protein MAEBL (PF3D7_1147800) in which alternative splicing of trans-membrane domain occurs [39]. Interestingly we found that the fourth exon of MAEBL, which includes a signal peptide, is skipped in one of the isoforms. The available RNA-seq studies [24,25,36,37] did not, however, report any form of alternative splicing in this gene. Experimental validation of the alternatively spliced exon coding for protein domains will be highly useful in determining their function effects. Some spliced exons coding for specific protein domains are conserved across species or result in transcript isoforms that are present in other species, implying that they may be functional. For example, we found alternative splicing of an ankyrin domain (PF3D7_0825100), homologous to human ankyrins that are known to occur in multiple isoforms [40].

### Assessing chip design principles

During our validation and use of the Nimblegen exon array, Nimblegen was purchased by Roche and subsequently the microarray platform was discontinued. This prompted us to investigate how well our exon array design principles would apply to other high-density microarray platforms. The Agilent HD exon array platform was chosen to test the transferability of design principles due to its high probe density, and the ease of platform customization to specific projects and hypotheses using the Agilent eArray online design software. Several factors determine the overall performance and reliability of an array including, the level of 3′ bias and probe hybridization intensity. Our exon arrays and sample preparation protocols have been optimized to overcome both of these potential pitfalls. By using the WTA2 cDNA synthesis kit (Sigma-Aldrich, US), we avoided 3′ bias in our sample preparations (S2 Fig). We calculated predicted probe binding intensities based on the frequency of dinucleotide combinations in the probe sequences (S3 Fig) which shows that TT rich probes are associated with very low hybridization intensities while GA rich probes have high intensities as has been previously documented by others [41]. Additionally, both of these factors can be further overcome by including a sufficient number of probes per gene on the array.

**Fig 5:**
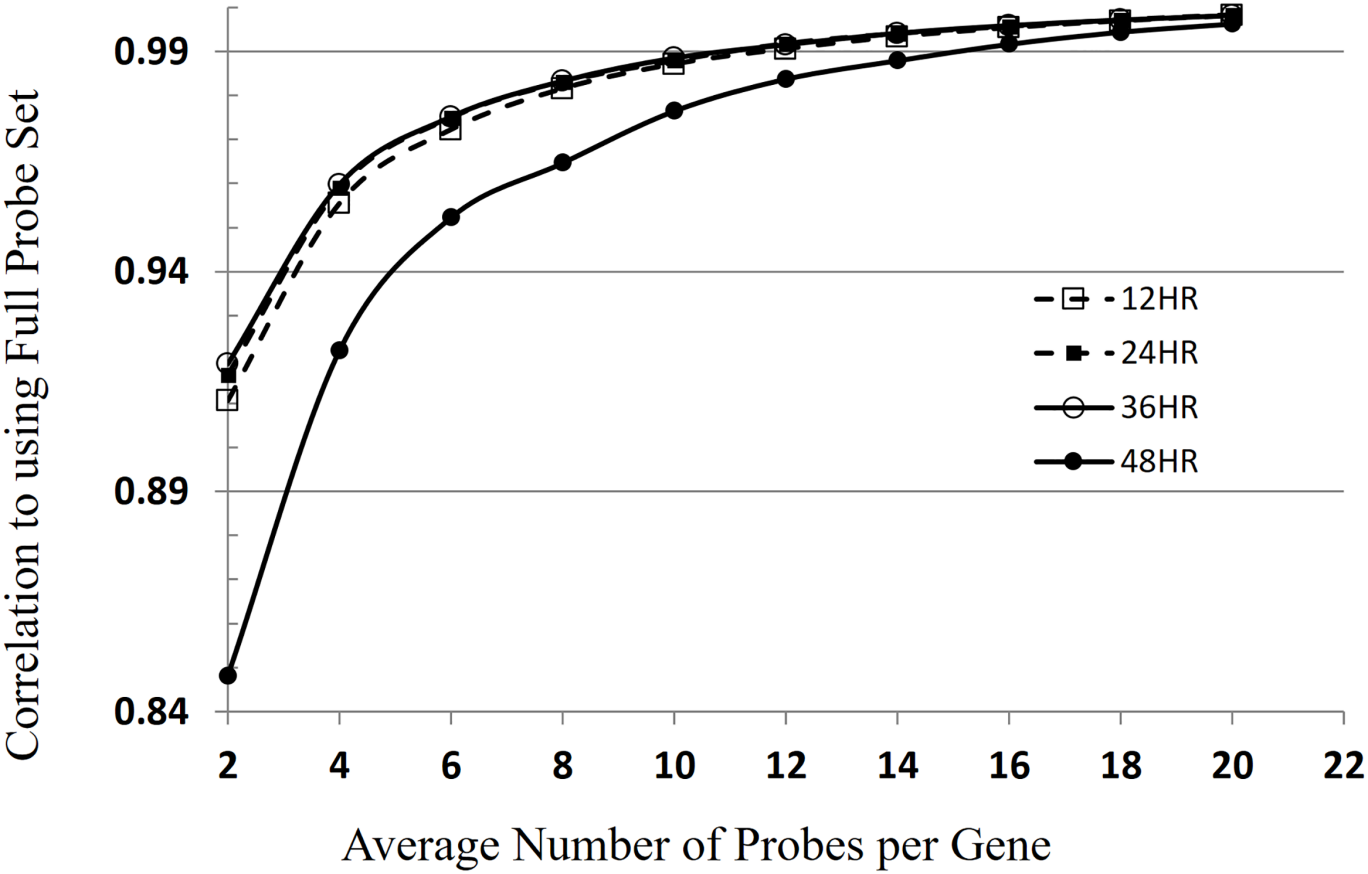
Determining the ideal number of probes per gene. Simulation of the minimum number of probes per gene required to reproduce gene expression levels obtained by using the full probe-set per gene on the exon array. For each time point, gene expression data was obtained by averaging signal intensity across a given subset of probes (2 to 20) and in each case the genome-wide correlation between the expression level obtained when using a subset of probes and the full probe set was determined. To obtain highly correlated signals between replicates, a minimum of 10–12 probes per gene is ideal.

To determine the optimal number of probes per gene for inclusion on the Agilent HD exon array, we simulated the average signal obtained from randomly generated subsets of 2–20 probes of those represented on the Nimblegen array platform. We then obtained the correlation between genome-wide expression levels for each subset to that obtained when using all probes present on the Nimblegen array for all genes on the array (5683 total genes compared) (Fig 5). As the number of probes in the subset grouping increased, the correlation increased up to a maximum of 0.99 for the subset containing 10 probes. Notably, this correlation varied somewhat by developmental stage. In particular, using a subset of probes, regardless of the number included, performed least well at the 48 hpi time point. This dependence could be influenced by the extended half-life of the mRNAs in schizonts (65 minutes, compared to the earlier stages (9 minutes in rings)) [42]. Importantly, as the number of probes in the subset increased to 10, the time-dependent effect diminished. Based on this analysis a minimum of eight probes per gene is ideal for adequate per gene coverage and signal consistency (95% correlation). These results support the value of high probe density but also demonstrate diminishing returns after probe density exceeds 12. We conclude that 8 to 12 probes per gene is a reasonable trade-off between performance and physical design constraints on other platforms. The Agilent HD exon array contains 62976 probe spots, and assesses 5440 genes with an average of 12 probes per gene.

## Discussion

Our analyses indicate that the transcriptional landscape of *P*. *falciparum* is complex and the routine monitoring of alternatively spliced mRNA and ncRNAs will be important to understand the significance to parasite development, response to drug, and environmental perturbations. While the future of microarrays remains hotly debated as RNA-seq becomes less expensive and efficiently analyzed, the results obtained here stress the need for exon-centric analyses of transcripts, including those obtained by RNA-seq. The data and design principles presented here demonstrate that microarrays have a niche in measuring whole transcription profiles. At less than $200 per sample, both the Nimblegen and Agilent HD exon arrays are cost-effective compared to RNA-seq for high throughput whole malaria transcriptome assessment of hundreds of samples. These platforms can be readily customized for hypothesis-driven investigations, can be quickly updated as new genome versions are released, and have well developed post-hybridization data pipelines for systems biology and network analyses [15,17].

Unlike other existing arrays for *P*. *falciparum*, the high-density exon arrays profile both transcript isoforms and ncRNAs. The array detects isoforms that are consistent with those previously reported by RNA-seq studies, and identifies potentially novel instances of alternative splicing. There is currently no comprehensive alternative splicing database for *P*. *falciparum* and, every new study has identified novel isoforms. The Agilent HD exon array identified many known alternate splicing events and predicted novel events. The identification of new alternative splicing on the exon array suggests there may be considerably more genes with variant isoforms than are currently curated in gene databases. Among the genes that were identified as alternatively expressed on the HD exon array but not in RNA-seq experiments, 39.8% had expression levels in the lowest quartile of genes measured by Otto *et al*. As the ability to detect alternative splicing using RNA-seq is dependent on the number of sequencing reads across a mRNA sequence, low expressing genes may not have produced enough sequencing reads in some of the cited studies to determine whether alternative splicing had occurred. Detection of novel splicing variants using microarrays is widely utilized in human genomic studies using the high-density arrays [27,43,44], and may be a valuable tool to assess gene isoforms in protozoan parasites as well. However, in the described experiments, there was no robust way to determine the proportion of false positives among our set of newly identified genes with alternative splicing. Thus, it will be important to continue to validate predicted isoforms and cross validate new data with published results.

Overall, the two exon arrays generate highly reproducible transcript abundance estimates using single-color platforms that are comparable to existing datasets collected using previous dual-color platforms. The high probe density of the arrays allow gene expression measurements for each gene to be estimated from a minimum of 8 probes across the entire mRNA length. The increased density enhances the reliability of the array by making it robust to sequence dependent probe hybridization biases and providing more reproducible transcript abundance measurements.

The protocol described in the methods uses a very low initial amount of RNA (300 ηg) which we can reliably extract from 10 mL of culture at 5% hematocrit and 1%—2% parasitemia. Even with these very low starting RNA volumes, the exon arrays demonstrated excellent reproducibility allowing for considerable resource use reductions. This protocol also allows samples to be stored long-term as unlabeled cDNA which has greater stability than RNA.

The new high-density exon arrays described provide new alternatives for Plasmodium expression profiling. The Agilent HD exon array, as a single-color platform, accurately measures mRNA abundance, ncRNA abundance and detects exon skipping.

## Materials and methods

### Design of the malaria exon arrays

The Nimblegen platform exon array [17] is a custom-designed microarray consisting of 12-plexes (12 ×135K format). On each plex are 128,179 features (probes) of 50 to 75-mers representing 5,683 annotated transcripts in PlasmoDB [45], ncRNAs [18] and novel transcripts [46]. For each transcript, exon sequences were downloaded from PlasmoDB (v 6.3). Exon sequences were binned into 3 categories depending on their length: i) exons longer than 1 kb were each targeted by 20 probes, ii) exons ranging from 200–1000 bases were each targeted by 10 probes and, iii) exons shorter than 200 bases were each targeted by 5 probes. For transcripts in which it was not possible to design a probe in all the exons, probe design was performed based on the full mRNA sequence. In addition to probes targeting transcripts, negative control probes, having no sequence similarity to the *P*. *falciparum* genome but of the same GC content and melting temperature as the transcript probes, were randomly generated and placed on the array.

The Agilent HD exon array is a custom-designed microarray consisting of 8-plexes (8 ×60K format). On each plex there are 62,976 features of 60-mers representing 5540 annotated transcripts, ncRNAs and novel transcripts. For each transcript, exon sequences were downloaded from PlasmoDB (v 9.3). Probes were designed 5′ to 3′ using the Agilent eArray online software and evenly spaced based on exon length such that: i) exons longer than 10 kb were targeted by one probe every 1000bp, ii) exons between 5–10 kb were targeted by one probe every 500bp, iii) exons between 1–5 kb were targeted by 10 probes spaced evenly throughout the exon, and iv) exons shorter than 1 kb were targeted by one probe every 100bp and these probes were duplicated on the array multiple times to provide a minimum of 8 probes per exon. Exons shorter than 45 bp were not included on the array. All probes were checked for hybridization quality, and cross-hybridization potential prior to inclusion on the array. In addition to probes targeting transcripts, negative and other control probes were placed randomly on the array using Agilent’s *P*. *falciparum* expression array control grid. Further information about the probe counts and distribution of probes across the genome is provided in S1 Methods.

### Parasite cultures and RNA extraction

The *P*. *falciparum* laboratory clone HB3 was grown using standard protocols [47]. Cultures obtained from a single parasite thaw were grown in replicates at 37°C and 5% hematocrit in O+ human red blood cells using RPMI 1640 (Invitrogen, Carlsbad, CA) supplemented with 0.5% Albumax I (Invitrogen, Carlsbad, CA), 0.25% sodium bicarbonate (Mediatech, Inc., Manassas, VA) and 0.01 mg ml-1 gentamicin (Invitrogen, Carlsbad, CA) under an atmosphere of 90% nitrogen, 5% oxygen, and 5% carbon dioxide. Cultures were synchronized using 5% sorbitol. Harvesting of cultures for RNA samples was performed at 12, 24, 36 and 48 hours post-invasion. Total RNA was extracted from 20mls of culture using TriZol reagent (Invitrogen, Carlsbad, CA) as described previously [48]. Quality and quantity of RNA was determined using Nanodrop (NanoDrop Technologies).

### cDNA synthesis, labeling and hybridization

300ηg of RNA was used as starting material for cDNA synthesis using the Sigma WTA2 whole transcriptome amplification kit as previously described [17].

1μg of cDNA was labeled with Cy3 dye using 65% AT rich pre-labeled random hexamers as primers for cDNA synthesis by Klenow fragment of DNA polymerase I. Hybridizations on the Nimblegen array were performed for 18 hours followed by washing of the arrays as described according to standard protocol (Roche NimbleGen Inc., Madison, WI). The microarray image was obtained using a 2μM scanner and probe intensity values extracted using NimbleScan software (Roche NimbleGen Inc., Madison, WI). For the Agilent HD exon array, hybridizations were performed for 17 hours. Images were obtained on the same 2uM scanner and probe intensity values extracted using Agilent Feature Extraction software.

### Gene expression analysis

Probe intensities were normalized using robust multichip average (RMA) method [49]. Normalization was performed across all samples hybridized on a single chip. Exon signal intensity for each gene was obtained by averaging the intensities of all probes within each exon. To determine a significance threshold for exon expression levels, a background distribution of signal intensities from a set of negative control probes (10,000 probes on the Nimblegen exon array and 1,000 on the Agilent HD exon array) with no sequence matches to the *P*. *falciparum* genome was generated. A threshold corresponding to the 95^th^ percentile (5% FDR) of the signal distribution of the negative control probes was then applied [27]. To determine gene expression levels, exons that passed the 5% significance threshold were subjected to an additional threshold derived from intensities of 1,000 simulated exons each consisting of 20 randomly sampled negative control probes. Intensities of exons that passed a 5% FDR based on this background distribution were averaged to obtain a gene expression average. More details are provided in the supplementary material. ncRNAs that showed high variation of fold-change ≥ 2 between replicates were excluded.

### Determination of concordance between platforms

Concordance between platforms was evaluated by comparing lists of differentially expressed genes obtained from each platform using replicates at 12 and 36 hour post-invasion. Genes with log_2_ ratio ≥ 2 or ≤ -2 (4-fold change) between 12 vs. 36 hpi samples were regarded as up-regulated and down-regulated, respectively.

The platforms were further compared using gene set enrichment analysis (GSEA) [32,50]; GSEA used ranked information to compare gene lists from independent studies. Given a query gene list ordered by confidence and a second rank ordered list of all genes (i.e. the gene set), GSEA represents the likelihood of finding the entries in the gene set at the top of the query list. For GSEA, four query lists were first constructed: up-regulated and down-regulated genes from the previously published Agilent 15K array, and up-regulated and down-regulated genes from the newly designed Agilent HD exon array. Genes from the Nimblegen exon array were sorted in descending order (from the most up-regulated to the most down-regulated) to make up the rank ordered gene set. The Broad GSEA online software was then used to query the gene lists from the both Agilent platform arrays against the ranked Nimblegen gene set.

### Transcript isoform analysis

Exon intensities meeting the 5% FDR criterion described above were considered for transcript isoform analysis. Alternative splicing was then estimated using two indices: i) normalized gene index (NI); and splicing index (SI), which have been used in transcript isoform analysis in Affymetrix exon arrays [51] where:

$$Normalized\;Gene\;Index,\;NI\;=\;\frac{Exon\;Level\;Expression}{Gene\;Level\;Expression}$$ (1)

$$Splicing\;Index=log2(\frac{Sample\;1\;NI}{Sample\;2\;NI})$$ (2)

An exon was considered to be alternatively spliced if the splicing index was ≥ 2 (indicating the exon is spliced out in the reference sample, sample 2) or ≤ -2 (exon spliced out in the target, sample 1). Exons whose expression was not detected in at least 2 replicates were excluded from the analysis.

Alternatively spliced exons were compared to those from RNA-seq studies [24, 25] using a hypergeometric test to determine the statistical significance of the overlaps of alternatively spliced exons detected by the exon array vs. RNA-seq based on the total number of genes in the genome containing multiple exons (N = 2781) [52]. Alternatively spliced exons were scanned for domains using PSSMs from the Conserved Domain Database [38] implemented in CD-Search [53].

### Agilent 15K array hybridization and data analysis

RNA obtained as described above at 12 and 36 hpi was prepared for hybridization using a validated protocol for cDNA synthesis from Dr. Manuel Llinas’ lab [20]. Labeling was performed using single strand aminoallyl-containing cDNA synthesis and Amersham CyDye coupling (GE Healthcare, Piscataway, NJ, USA). cDNA for the two developmental stages was labeled using Cy5 and hybridized to an equal amount of Cy3-labeled cDNA reference pool obtained from equal amounts of rings, trophozoites and schizonts. Hybridization was performed on printed 8 X 15K arrays ordered from Agilent (Agilent Technologies, Santa Clara, CA, USA) for 16 hours in a rotating hybridization oven (10 rpm) at 65°C. Arrays were scanned using Agilent G25OB Microarray Scanner at 5 μm resolution at wavelengths of 532ηm (Cy3) and 633ηm (Cy5) using the extended dynamic range setting (10–100%). Normalized signal intensities were extracted using Agilent feature Extractor Software Version 9.5 and further analysis performed on the Princeton University Microarray Database (PUMA.princeton.edu).

### Determining optimum number of probes

Data from the Nimblegen exon array was used to determine the optimum number of probes per gene that should be included on the Agilent HD exon array. Of the total probes on the Nimblegen array, the signal was averaged across probes selected in randomly generated even-numbered subsets of the total number of probes per gene ranging from subsets of 2 probes to 20 probes per gene. The average signal intensity for one subset of each number (2, 4, 6 …20) was generated for each gene in the genome. These randomly generated subsets were then correlated to the average signal intensity of all probes per gene on the array.

## Supporting information

S1 Table Comparison of up-regulated genes on the Nimblegen, Agilent HD exon, and Agilent 15K arrays. All genes that were up-regulated on any array are represented in column A. The “X” symbol is used to indicate in which array and replicate the gene was found to be up-regulated.
(CSV)

S2 Table Comparison of down-regulated genes on the Nimblegen, Agilent HD exon, and Agilent 15K arrays. All genes that were down-regulated on any array are represented in column A. The “X” symbol is used to indicate in which array and replicate the gene was found to be down-regulated.
(CSV)

S3 Table Comparison of ncRNA detected on the Nimblegen and Agilent HD exon arrays, and via Northern blot. The Gene ID for each ncRNA is provided in column A and the RNAz_ID in column B. The “X” symbol indicates whether the ncRNA was detected on the arrays or by Northern blot [18]. The sequence of the ncRNA is also listed.
(XLSX)

S4 Table Differential expression of ncRNAs detected on the Agilent HD exon array. The Gene ID for each ncRNA is provided in column A and the RNAz_ID in column B. The normalized fluorescent intensity values of each differentially expressed ncRNA at the corresponding time point for HB3 is provided in columns C-F, and the differential expression between time points are represented as log_2_ ratios in columns G-L.
(XLSX)

S5 Table Alternative splicing events identified on the Agilent HD exon array and comparison to RNA-seq studies. The Gene ID and exon number are given in columns A and B. Superfamily IDs and any conserved protein domains identified in the CDSS search are given in columns C and D. The PlasmoDB description of the gene is listed in column E. Averaged normalized fluorescent intensity values per exon on the Agilent HD array at the indicated time point are provided in columns F-I, and the averaged intensity for all probes within the entire gene are listed in columns K-M. NI values for the time point are given in N-Q, and SI values are in columns R-W as described in the supplement. Columns X—AA indicate by the “X” which other references or arrays detected alternative splicing.
(XLSX)

S1 Fig Distribution of probes throughout the transcriptome. Histogram distributions for the number of probes per gene (A and B) and base pair distance between probes (C and D) on the Nimblegen exon and Agilent HD exon array.
(TIF)

S2 Fig Signal intensity is consistent among exons within a transcript. The relationship between the signal intensity for annotated exons and the location of the exons within the gene demonstrates that the signal distribution of hybridized samples is on average of similar intensity from the most 5′ exon (exon 1) to the most 3′ exon (exon 5).
(TIF)

S3 Fig Effect of probe sequence on hybridization signal intensity. (A) Correlation between the frequency of each dinucleotide and the observed signal intensity of a probe.1^st^ base refers to the first nucleotide in the dinucleotide considered and second base to the second nucleotide in the same dinucleotide. (B) Correlation between the actual signal intensity of a set of probes to their predicted intensity based on a linear model of dinucleotides constructed from independent set of probes. The predictability of signal intensity from probe sequence alone (r = 0.56) signifies that the measurement of gene expression from a single probe cannot adequately be predicted from the during the design process. Robust gene expression measurements require expression level of a single gene to be determined from multiple independent probes.
(TIF)

S1 Methods Supplementary methodology and rationale for high density gene expression array designs. Processes and rationale for unbiased cDNA synthesis, and balanced design using sequence dependent hybridizing probes, along with more in depth technical explanations of the gene expression and transcript isoform analyses are described.
(DOCX)
