# Supplementary figures and images for: Simultaneous genome-wide gene expression and transcript isoform profiling in the human malaria parasite
Source: PLoS One. 2017 Nov 7;12(11):e0187595. doi: 10.1371/journal.pone.0187595 (PMC5675406; doi:10.1371/journal.pone.0187595)

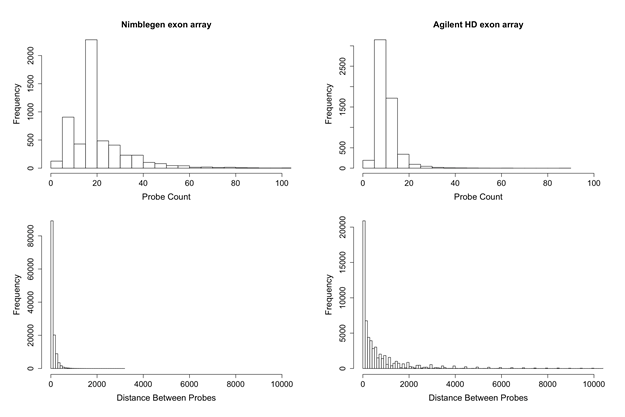

Supplement: S1 Fig — Histogram distributions for the number of probes per gene (A and B) and base pair distance between probes (C and D) on the Nimblegen exon and Agilent HD exon array. (TIF) [file pone.0187595.s006.tif]

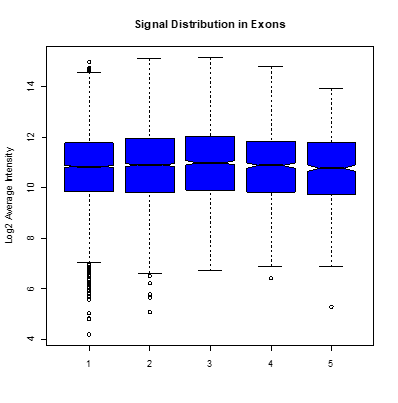

Supplement: S2 Fig — The relationship between the signal intensity for annotated exons and the location of the exons within the gene demonstrates that the signal distribution of hybridized samples is on average of similar intensity from the most 5′ exon (exon 1) to the most 3′ exon (exon 5). (TIF) [file pone.0187595.s007.tif]

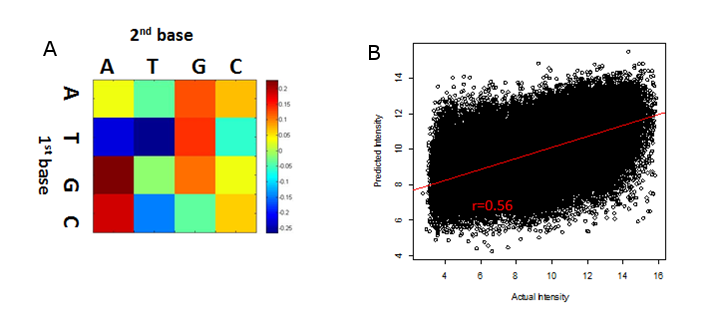

Supplement: S3 Fig — (A) Correlation between the frequency of each dinucleotide and the observed signal intensity of a probe.1st base refers to the first nucleotide in the dinucleotide considered and second base to the second nucleotide in the same dinucleotide. (B) Correlation between the actual signal intensity of a set of probes to their predicted intensity based on a linear model of dinucleotides constructed from independent set of probes. The predictability of signal intensity from probe sequence alone (r = 0.56) signifies that the measurement of gene expression from a single probe cannot adequately be predicted from the during the design process. Robust gene expression measurements require expression level of a single gene to be determined from multiple independent probes. (TIF) [file pone.0187595.s008.tif]
