# Supplementary material for: Simultaneous genome-wide gene expression and transcript isoform profiling in the human malaria parasite
Source: PLoS One. 2017 Nov 7;12(11):e0187595. doi: 10.1371/journal.pone.0187595 (PMC5675406; doi:10.1371/journal.pone.0187595)
Supplement: S1 Methods — Processes and rationale for unbiased cDNA synthesis, and balanced design using sequence dependent hybridizing probes, along with more in depth technical explanations of the gene expression and transcript isoform analyses are described. (DOCX) [file pone.0187595.s009.docx]

**S1 Methods**

**Unbiased cDNA synthesis**

The exon array consists of probes that target the whole span of segments of a gene from the most 3′ end to the most 5′ end. It is therefore important to determine whether the cDNA synthesis protocol used (WTA-2, Sigma-Aldrich) gives an unbiased coverage of exon sequences. To test this, we obtained total RNA from a mixed culture of the reference *P. falciparum* laboratory clone 3D7. cDNA synthesis was then performed using the WTA-2 kit (Sigma-Aldrich). The cDNA was then labeled with Cy-3 dye as explained in the Methods section. Hybridization was performed on a custom-designed whole genome tiling array [1]. We then examined the relationship between the signal intensity for annotated exons and the location of the exons within the gene (S2 Fig). It can be observed that the signal distribution of hybridized samples is on average of similar intensity from the most 5′ exon (exon 1) to the most 3′ exon (exon 5).

**Sequence Dependent Probe Hybridization Intensity**

Probe hybridization intensity is influenced by at least 2 main factors: i) the amount (copy number) of the corresponding labeled cDNA, and ii) the nucleotide sequence:

$$I\text{p}=\alpha C+\gamma S+\varepsilon$$

Where *I_p_* is the observed intensity of a given probe, $\alpha\text{and} \gamma$ are coefficients representing the contribution of copy number, *C*, and the sequence dependent effects, *S*, respectively. $\varepsilon$ represents random errors or other effects that cannot be explained by copy number or sequence specific effects.

Therefore, to model sequence dependent probe effects, we hybridized genomic DNA from the HB3 clone, under the assumption that the vast majority of genomic DNA segments occur in equimolar concentrations. Hybridization was performed on the Malaria exon array. Since many physical properties of DNA including sequence specific hybridization [2] and melting kinetics [3, 4]captured by dinucleotides, we applied multiple linear regression to determine the relationship between the dinucleotide composition of probes and the signal intensity observed from hybridization of genomic DNA:

$$log\text{2}(I)= \sum_{i=1}^{16} \beta\text{i}A\text{i}+ \varepsilon$$

Where *I* is the actual probe intensity, *i* represents the 16 possible dinucleotides, *A_i_* is the frequency of the *i^t^*^h^ dinucleotide and $\varepsilon$ represents randomly distributed errors. For simplicity, this model does not take into account the possible position specific dinucleotide effects. For example, the occurrence of AA at the first position in 25-mer probes of affymetrix arrays has a different effect from the occurrence of the same dinucleotide in the middle of the probe [5]. Probes on the exon array are at least twice as long as affymetrix probes (50 to 75-mers on exon array compared to 25-mers on affymetrix). This implies that if position specific effects are included, the amount of model parameters to be considered would increase from 16 to 800 (16 dinucleotides x 50 nucleotide positions) for a 50-mer probe. This would lead to overfitting.

To learn the parameters of this model from data, 66% of observed probe hybridization intensity data was used to build a linear model in the WEKA machine learning package. The hybridization intensity of the remaining probes was then predicted using the linear model. The correlation between the actual and predicted intensities was 0.56 (S3 Fig). A similar analysis of the sequence dependent fluorescent intensity of microarrays using cyanine dyes found similar increases in predicted and actual signal with increasing numbers of dG per probe [6] and further underscores the importance of using multiple probes to interrogate each exon.

**Gene Expression Analysis**

Given that for each gene the exon array has multiple probes, we computed overall expression measurements using both the exon level mean signal intensity, and a mean gene expression level using the average intensity across all exons using background corrected, RMA normalized intensities in NimbleGen SignalMap sofware.

RMA normalized data was used to obtain gene expression levels as explained below:

1. Exon Level signal intensity

- To obtain exon level signal intensity, a background distribution of signal intensity from a set of negative control probes with no sequence similarity to the *P. falciparum* genome was used to determine a threshold of significance. These control probes were then sorted in descending order (from the one with the highest signal to that with the lowest signal).
- To determine a 5% false discovery rate (FDR), the 50^th^ highest signal intensity of the negative control probes was taken as the cut-off for a probe to be considered as significantly expressed. Any probes that had signal intensity less than the 51^st^ highest signal in the negative control probe set was excluded from downstream analysis. This signal cut-off corresponded to the 95^th^ percentile of the signal distribution of the control probes and was conceptually similar to the detection above background (DABG) method by Affymetrix [7, 8].
- An exon level expression intensity was then obtained by averaging the signal of all probes targeting an exon, considering only probes whose intensity passed the 5% FDR threshold or the data dependent threshold described above.

1. Gene Level Signal Intensity

- The expression level for each gene was determined by averaging the exon level intensities obtained by the procedures outlined in (i), considering only exons that passed an additional filtering criteria described below.
- To determine significantly expressed exons, a background distribution of ‘simulated exon’ signals was estimated by first sampling 20 negative control probes randomly, repeating this 1000 times and each time computing the average signal intensity.
- The signal distribution of the simulated exons was then used to determine a 5% FDR signal cut-off by taking the 95^th^ percentile of their signal intensity as the cut-off.
- Exons whose signal intensity passed this threshold were then considered as significantly expressed.
- The gene level signal intensity was then taken as the average intensity of significantly expressed exons targeting the gene.
- FDR was also be obtained for individual exons simply as the proportion of simulated exons whose signal intensity exceeded the observed intensity for the given exon.

**Alternative Splicing/ Transcript Isoform Analysis**

The basis of the alternative splicing analysis is that the signal intensity of an alternatively spliced exon should be significantly lower when compared to the gene as a whole. Only exons and genes whose signal intensity have passed the cut-offs in (B) were considered for this analysis.

- In order to determine whether a gene is alternatively spliced in any two development stages, we first computed two indices- the normalized gene index (NI) and the splicing index (SI). Both indices have been used in detection of alternative splicing in affymetrix human exon arrays [8]. The gene index is the ratio between the expression level of an exon to the expression level of the gene.

$$Normalized Gene Index, NI=\frac{Exon Level Expression}{Gene Level Expression}$$

- Given two samples, the splicing index (SI) for a given exon is the ratio of the normalized gene index in sample 1 (NI) to that in sample 2 (NI).

$$Splicing Index=log2\left( \begin{aligned} \frac{Sample 1 NI}{Sample 2 NI} \\ \end{aligned} \right)$$

- A splicing index of 0 implies that the exon is equally included in both samples while an index greater than 1 indicates that the exon is enriched in sample 1. On the other hand, splicing index less than 1 shows that exon skipping is higher in sample 1 than in sample 2.

References

 1. Samarakoon, U., Gonzales, J. M., Patel, J. J., Tan, A., Checkley, L., & Ferdig, M. T. (2011). The landscape of inherited and de novo copy number variants in a plasmodium falciparum genetic cross. *BMC Genomics, 12*, 457-2164-12-457. doi:10.1186/1471-2164-12-457; 10.1186/1471-2164-12-457.

2. Naef, F., & Magnasco, M. O. (2003). Solving the riddle of the bright mismatches: Labeling and effective binding in oligonucleotide arrays. *Physical Review.E, Statistical, Nonlinear, and Soft Matter Physics, 68*(1 Pt 1), 011906.

3. Breslauer, K. J., Frank, R., Blocker, H., & Marky, L. A. (1986). Predicting DNA duplex stability from the base sequence. *Proceedings of the National Academy of Sciences of the United States of America, 83*(11), 3746-3750.

4. SantaLucia, J.,Jr, Allawi, H. T., & Seneviratne, P. A. (1996). Improved nearest-neighbor parameters for predicting DNA duplex stability. *Biochemistry, 35*(11), 3555-3562. doi:10.1021/bi951907q.

5. Gharaibeh, R. Z., Fodor, A. A., & Gibas, C. J. (2008). Background correction using dinucleotide affinities improves the performance of GCRMA. *BMC Bioinformatics, 9*, 452-2105-9-452. doi:10.1186/1471-2105-9-452; 10.1186/1471-2105-9-452.

6. Agbavwe, C., and Somoza, MM. (2011). Sequence-Dependent Fluorescence of Cyanine Dyes on Microarrays. *PLOSONE,* doi:10.1371/journal.pone.0022177.

7. Bemmo, A., Benovoy, D., Kwan, T., Gaffney, D. J., Jensen, R. V., & Majewski, J. (2008). Gene expression and isoform variation analysis using affymetrix exon arrays. *BMC Genomics, 9*, 529. doi:10.1186/1471-2164-9-529

8. Clark, T. A., Schweitzer, A. C., Chen, T. X., Staples, M. K., Lu, G., Wang, H., *et. al.* (2007). Discovery of tissue-specific exons using comprehensive human exon microarrays. *Genome Biology, 8*(4), R64. doi:10.1186/gb-2007-8-4-r64.
